# Identification and characterization of host miRNAs that target the mouse mammary tumour virus (MMTV) genome

**DOI:** 10.1098/rsob.240203

**Published:** 2024-12-11

**Authors:** Bushra Gull, Waqar Ahmad, Jasmin Baby, Neena G. Panicker, Thanumol Abdul Khader, Tahir A. Rizvi, Farah Mustafa

**Affiliations:** ^1^ Department of Biochemistry & Molecular Biology, College of Medicine & Health Sciences (CMHS), United Arab Emirates University, Al Ain, UAE; ^2^ Department of Microbiology & Immunology, College of Medicine & Health Sciences (CMHS), United Arab Emirates University, Al Ain, UAE; ^3^ Zayed Center for Health Sciences (ZCHS), United Arab Emirates University, Al Ain, UAE; ^4^ ASPIRE Research Institute in Precision Medicine, Abu Dhabi, UAE

**Keywords:** mouse mammary tumour virus (MMTV), breast cancer, host–virus interactions, miRNA target prediction, miRNA–mRNA docking, gene ontology and KEGG pathways

## Abstract

The intricate interplay between viruses and hosts involves microRNAs (miRNAs) to regulate gene expression by targeting cellular/viral messenger RNAs (mRNAs). Mouse mammary tumour virus (MMTV), the aetiological agent of breast cancer and leukaemia/lymphomas in mice, provides an ideal model to explore how viral and host miRNAs interact to modulate virus replication and tumorigenesis. We previously reported dysregulation of host miRNAs in MMTV-infected mammary glands and MMTV-induced tumours, suggesting a direct interaction between MMTV and miRNAs. To explore this further, we systematically examined all potential interactions between host miRNAs and the MMTV genome using advanced prediction tools. Leveraging miRNA sequencing data from MMTV-expressing cells, we identified dysregulated miRNAs capable of targeting MMTV. Docking analysis validated the interaction of three dysregulated miRNAs with the MMTV genome, followed by confirmation with RNA immunoprecipitation assays. We further identified host targets of these miRNAs using mRNA sequencing data from MMTV-expressing cells. These findings should enhance our understanding of how MMTV replicates and interacts with the host to induce cancer in mice, a model important for cancer research. Given MMTV’s potential zoonosis and association with human breast cancer/lymphomas, if confirmed, our work could further lead to novel miRNA-based antivirals/therapeutics to prevent possible MMTV transmission and associated cancers in humans.

## 1. Introduction

The mouse mammary tumour virus (MMTV) is a *betaretrovirus* that causes breast cancer in mice [1,2]. The virus is transmitted from infected mothers to the progeny through breast milk that contains high levels of virus particles [3]. Upon ingestion by the nursing pups, the gut-associated lymphoid tissues are the first ones to get infected, including dendritic cells, B cells and T cells [1,2]. These infected B and T cells circulate in the body and spread the virus to highly susceptible mammary epithelial cells, the main targets of virus replication and tumorigenesis. Infection of the mammary gland leads to enhanced virus replication in mammary epithelial cells as mice mature during puberty, pregnancy and lactation. This process ultimately leads to mammary tumours due to insertional mutagenesis upstream of growth-promoting genes, including *Wnt*, *Fgf*, *Notch4* and *Rspo* [4]. MMTV is also observed in an endogenous form, passaged vertically to the progeny as an integrated provirus within the genome (reviewed in [1,5]). Found in most wild-type and laboratory-bred mice, most such endogenous viruses (termed ‘*mtvs*’) are defective, and are unable to cause cancer due to mutations in their structural genes, except for a few strains that are replication-competent.

The MMTV genome encodes several canonical structural (*gag* and *env*) and enzymatic (*pro* and *pol*) genes present in all retroviruses, along with specialized accessory/regulatory genes (*dut*, *rem* and *sag*). These genes are important for virus replication, gene expression and its spread within mice, respectively [2]. The MMTV genome is flanked by long terminal repeats (LTRs) that possess numerous *cis*-acting regulatory elements that control viral gene expression, such as promoters, transcription factor binding sites and mammary gland enhancer, hormone-responsive and negative regulatory elements, as well as sequences essential for virus replication [2]. The MMTV LTR is unique in that it encodes an entire open reading frame (*sag*) within its LTR, unlike most other retroviruses. Mouse models driven by the MMTV LTR have garnered significant attention and use within the scientific community for investigating the intricate process underlying breast cancer initiation, development and progression. This is largely due to the discovery of oncogenes using this model system that play important roles in both mouse and human breast cancer [1,4].

Viruses and host cells are constantly engaged in a struggle for survival using multiple strategies, including RNA interference (RNAi), a gene-silencing mechanism used by hosts and viruses crucial in regulating various cellular processes, including viral infections. In the case of eukaryotes, various small RNAs have evolved to fight viral infections, silence unnecessary gene transcripts originating from transposable elements and regulate host gene expression [6]. MicroRNAs (miRNAs), P-elementinduced-wimpy testis (PIWI)-interacting RNAs (piRNAs) and small interfering RNAs (siRNAs) are three categories of small RNAs that can be found in animals, ranging from 20 to 35 nucleotides (nt). In most tissues, miRNAs are the predominant type of small RNAs involved in every aspect of host cell development and function [6–8]. They are important gene expression modulators via RNA silencing or post-transcriptional modifications [6,7]. miRNAs play crucial regulatory roles in various cellular processes, such as proliferation, differentiation, apoptosis and cell cycle regulation, as well as disease induction, including initiation and invasion/metastasis of cancer [6–8]. They accomplish this by deadenylating and degrading messenger RNA (mRNA) transcripts or inhibiting their translation [6,7].

The biogenesis of miRNAs begins in the nucleus where they are encoded within the genome as normal genes that code for proteins. Most cellular and viral miRNAs (v-miRs) are initially produced as pri-miRNAs hundreds to thousands of nucleotides long with at least one or more approximately 80 nucleotide stem-loop structures (reviewed in [6–10]). Like mRNAs, miRNAs are capped and polyadenylated and further processed into approximately 65–70 nucleotide pre-miRNAs in the nucleus, which are then exported to the cytoplasm through nucleopores where they are further processed by Dicer into fully mature approximately 21–24 nucleotide miRNAs that now resemble siRNAs of the RNAi pathway. The two strands are then loaded onto the RNA-induced silencing complex (RISC) that contains the slicer protein, Argonaute (AGO), and other cofactors [11]. AGO, with the help of the RISC-associated proteins, helps unwind the double-stranded miRNA and selects one strand for presentation to the cellular mRNAs, while the other ‘passenger strand’ is degraded. The selected miRNA ‘guide strand’ then targets specific mRNAs via complementarity, which can number in hundreds. If the complementarity is perfect between the two molecules, the mRNA is destabilized and degraded (as happens mostly in plants), while if there is imperfect base pairing, the mRNA undergoes translational inhibition, as is observed mostly in animal. Thus, RISC serves as an active core where specific cellular or viral mRNAs are targeted through incomplete Watson–Crick base-pairing by the miRNAs produced by the animal cells. The phenomenon of particular targeting is observed via the seed region, located at the 5′ end of the miRNA, starting from the second nucleotide in the mature miRNA, which is 6–8 nucleotides in length [6,7,12–17]. This region pairs with a particular sequence of the target mRNA called the miRNA response element (MRE). Most cellular genes contain an evolutionarily conserved MRE, primarily found in the 3′-untranslated region (UTR) of genes. However, several studies have shown that miRNAs can also interact with 5′ UTRs and coding sequences to regulate gene expression [18–21].

It is generally thought that ‘seed’ binding should be fully maintained for stable miRNA−mRNA heteroduplex formation. However, recent studies are suggesting that 1–2 wobble base pairings may be tolerated within the seed and miRNA binding, depending upon other factors, such as stability of the miRNA−mRNA heteroduplex, the ‘accessibility’ of the miRNA binding site on the mRNA, as well as the level of conservation of the miRNA/mRNA sequences [22,23]. Thus, a few changes in traditional base-pairing and binding site specificity could make it hard to determine how a target is recognized [24]. Interestingly, transcriptomics analyses have the capability to provide information on the number and the spatial distribution of MREs associated with a specific miRNA within the 3′ UTR [20]. The degree to which MREs are conserved across species can be estimated by looking at the evolutionary conservation of their sequences [25]. Potential miRNA–target mRNA interactions can be identified using their binding metrics with greater consistency and accuracy. The regulatory functions of miRNAs can only be predicted in their entirety through an understanding of potential target recognition mechanisms [12,13]. For this reason, miRNA target prediction has thus far relied primarily on *in silico* methods prior to experimental verification [26–29].

Over the past decade, numerous studies have focused on a comprehensive evaluation of miRNA expression, describing substantial changes in their expression profiles in various pathologies, including psoriasis, arthritis, fibrosis and cancer [30–34]. Due to their target specificity, miRNAs have also become an indispensable tool in disease diagnosis as well as in therapy [32–34]. Furthermore, miRNAs play crucial roles in modulating viral infections that can have both antiviral or proviral effects [35–37]. For example, viral infections can lead to the induction of antiviral host miRNAs involved in the innate cellular response to the viral infection. Host miRNAs can also directly block infection or suppress host factors that facilitate viral infection. On the other hand, viruses can exploit miRNAs to their advantage and initiate a proviral response to counteract the host defence systems and/or facilitate their own replication, persistence or infection. They can do that by inhibiting the maturation or increasing the degradation of host antiviral miRNAs, inhibiting cellular factors that facilitate viral infections, or directly interacting with viral genomes to facilitate their replication [35–38]. Some viruses, especially DNA viruses, can further manipulate the host response to the viral infection by encoding v-miRs, though RNA viruses can also encode them [39–41]. These v-miRs participate in the virus life cycle to facilitate viral replication and help establish latency. At the same time, v-miRs can affect important cellular pathways, including cell cycle regulation, apoptosis, oncogenesis and evasion of the virus from host immune response [35,36]. For example, they can mimic cellular miRNAs that induce immune suppression and lymphocyte proliferation [42].

One way by which host miRNAs can play both antiviral and proviral roles is via their ability to target the viral genome, an observation made in several viral infections, including human immunodeficiency virus (HIV), hepatitis C virus (HCV), bovine viral diarrhoea virus (BVDV) and others [30–32]. We have previously demonstrated that MMTV does not encode v-miRs; instead, it induces dysregulation of host miRNAs, increasing the expression of some miRNAs, while downregulating the expression of others [43]. In the current study, several bioinformatic tools were used to examine all possible interactions between host miRNAs and the MMTV genome, followed by their deeper analysis via miRNAseq data from MMTV-expressing cells, resulting in the identification of three miRNAs that could target the MMTV genome. This observation was further supported by miRNA−mRNA docking analysis and RNA immunoprecipitation (RIP) assays. In addition, we elucidated the potential mRNA targets and cellular pathways of these miRNAs and how they may regulate MMTV replication and affect host gene expression using our previously published transcriptome data from MMTV-expressing cells [44]. These results provide valuable insights into the molecular relationship between MMTV and its host, which may ultimately facilitate a better understanding of virus replication within mammary epithelial cells and spread in the host, as well as how the virus induces mammary carcinogenesis in mice.

## 2. Methods

A number of tools were employed in this study based on their core algorithms. These computational methods involved seed specificity/match, conservation of target positions, target interactions in predicted transcripts, comparison of results with gene expression data obtained from normal and virus-expressing cells as well as experimentally validated targets, and analysis of binding sites on a given transcript along with their associated binding energy using docking tools. The binding of MMTV RNAs to RNAi machinery (RISC) was confirmed via immunoprecipitation assays. Finally, the target genes of the shortlisted miRNAs were predicted and analysed for the cellular pathways they affect.

### 2.1. MMTV genome sequence

The MMTV sequences were retrieved from the National Centre of Biological Information with the accession number AF228552.1 representing the C3H strain of MMTV (https://www.ncbi.nlm.nih.gov/nuccore/AF228552.1).

### 2.2. RIP assays

RNA immunoprecipitation (RIP) assays were conducted using cell lysates from control HEK293T cells and HEK293T cells stably expressing MMTV [45]. HEK293T are human cells that do not contain endogenous MMTV strains (*Mtvs*) that could have confounded our results. RIP assays were performed using the miRNA Target IP kit (Active Motif, Carlsbad, CA, USA) as directed by the manufacturer with slight modifications. First, the pan-AGO antibody was conjugated to the protein G beads after pre-incubation with a non-specific protein like bovine serum albumin to remove background. A 100 µl aliquot of the cell lysate was mixed with protein G magnetic beads and immunoprecipitated with 5 µg of either anti-pan-AGO or isotype-specific control anti-IgG antibody in 900 µl of immunoprecipitation buffer overnight at 4°C. The samples for RNA isolation were subjected to proteinase K treatment to facilitate the release of proteins from RNA, followed by RNA isolation from beads using phenol/chloroform/isoamyl alcohol (25 : 24 : 1) extraction, as described in the kit. The isolated RNAs were resuspended in RNase-free water and processed for cDNA synthesis and RT-PCR analysis, as described earlier [46]. Another aliquot of the cell lysate before and after immunoprecipitation was saved for Western blot analysis without proteinase K digestion, performed as described earlier [45].

### 2.3. Prediction of miRNA target sites

To evaluate the robustness, precision and accuracy of the potential miRNA−mRNA interactions, target prediction tools compute the minimum free energy (MFE) at a specific energy level [47]. Therefore, we used four stringent target prediction tools in this study, including miRanda (http://www.microrna.org/microrna), RNAhybrid (https://bibiserv.cebitec.uni-bielefeld.de/rnahybrid), STarMir (https://sfold.wadsworth.org/cgi-bin/starmirWeb.pl) and RNA22 v. 2.0 (https://cm.jefferson.edu/rna22/). All four programs were run at default settings while keeping the MFE value Δ*G* = −20.0. Targeted miRNAs were predicted using the first three tools and then these were matched with RNA22 predictions. Only those miRNAs were selected that were predicted with all four tools with the specified free energy.

### 2.4. Validation of the expression of the predicted miRNAs and mRNAs

RNAseq for mRNA was performed on control and MMTV-expressing BLAB/c mouse mammary epithelial HC11 cells, as previously described [44]. miRNAseq was performed on the same RNA samples in parallel. Briefly, total RNA was extracted from control and MMTV-expressing HC11 cells using the TRIzol reagent (Thermo Fisher Scientific). In addition to mRNAseq, isolated RNA from two independent biological controls was commercially sequenced for miRNAseq by the Beijing Genomics Institute (Hong Kong) using their proprietary DNBSEQ (DNA nanoball-based sequencing) technology employing unique molecular identifiers, resulting in reduced amplification errors and lower rates of duplicate counts, making the miRNAseq much more reproducible than counts based only on reads only (https://www.bgi.com/global/science-detail/small-rna-sequencing). Bowtie2 was used for read alignments to the host genome, while DEGSeq (Differentially expressed genes from RNA-seq data) was used to look for differentially regulated miRNAs. The miRNAseq data (BioProject accession number: PRJNA1000922) and mRNAseq data (BioProject accession number: PRJNA915407), both in their raw and analysed forms, are available for download from the server. These datasets can be utilized for data reanalysis and subsequent processing.

### 2.5. 2D/3D structure predictions of mRNA and miRNAs and docking of miRNA and mRNA

The miRbase database [48] was used to confirm the miRNA sequences used in this study. The MMTV DNA sequences were converted to RNA using the online tool Transcription & Translation (https://biomodel.uah.es/en/lab/cybertory/analysis/trans.htm). These were further used to predict the secondary and tertiary structures of miRNAs used during the docking analysis. RNAStructure v. 6.4 from Mathews and colleagues [49] was used to create two-dimensional (2D) structures of miRNAs and mRNAs in this study. RNAComposer (https://rnacomposer.cs.put.poznan.pl/) was used to predict the three-dimensional (3D) structures of the mRNA using the default parameters [50]. However, RNAComposer can only predict a 3D structure of an RNA for up to 500 nt long. To dock the miRNAs with mRNAs, 3D structures were submitted to the HNADOCK server (http://huanglab.phys.hust.edu.cn/hnadock/). The HNADOCK server uses a hierarchical algorithm (fast Fourier transform)-based global search strategy to predict the binding structure between two nucleic acids in the form of 3D models/pdb files [51]. The bonding patterns of the predicted models were visualized using UCSF Chimera v. 1.14 [52].

### 2.6. Prediction of possible targeted mRNAs and GO analysis

Several methods are available to predict targets of miRNAs that are either supervised or semi-supervised based on a support vector machine. Most of these servers predict miRNA−mRNA interactions based on high-throughput sequencing experiments or other verified sources. We employed four commonly used tools, including miRWalk (http://mirwalk.umm.uni-heidelberg.de/) [53], miRDB (https://mirdb.org/) [54], TargetScan (https://www.targetscan.org/vert_80/) [13] and MR-microT (https://mrmicrot.imsi.athenarc.gr/) to predict targets for the miRNAs identified in this study [55]. The accumulated results from all servers were further confirmed by mRNAseq analysis. The set of genes exhibiting differential regulation, commonly referred to as ‘differentially regulated genes' (DEGs), was submitted to the DAVID bioinformatics resource (https://david.abcc.ncifcrf.gov/tools.jsp). This platform was utilized for the analysis of Gene Ontology (GO) and Kyoto Encyclopedia of Genes and Genomes (KEGG) pathways, using *Mus musculus* as the reference species.

## 3. Results

### 3.1. Experimental strategy

**Figure 1.**
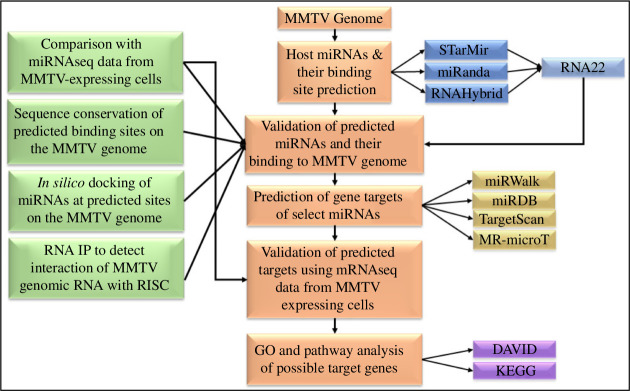
Experimental design to identify and characterize the host miRNAs that target the MMTV genome. The overall strategy followed in this study is outlined, starting with the MMTV genome from the C3H strain of MMTV. The orange boxes represent the main investigation pipeline that used previously published mRNAseq data from MMTV-expressing cells [44] and various bioinformatic tools listed on the right in blue, gold and purple boxes. The green boxes to the left illustrate the steps used to characterize the resulting miRNAs using the previously published miRNAseq data from MMTV-expressing cells [45], conservation of the binding sites predicted, docking analysis of the predicted miRNA sites on the MMTV genome and RIP assays.

To identify host miRNAs that can target the MMTV genome, a multipronged strategy was undertaken, as shown in figure 1. As a first step, several bioinformatic tools were used to predict the ability of host miRNAs to bind to the MMTV genome. The predicted miRNAs were then shortlisted using our miRNAseq data generated from MMTV-expressing mammary epithelial HC11 cells. The binding sites of the select top three miRNAs on the MMTV genome were then characterized for their conservation across MMTV strains and their ability to dock to their target sites on the MMTV genome. This was followed by testing the ability of MMTV genomic RNA to be targeted by the AGO proteins of the RISC, using RIP assays (figure 1). In the next phase of the study, we focused on the host gene targets of these miRNAs. First, we predicted which host mRNAs could be the potential targets of the selected miRNAs and analysed their expression in the same MMTV-expressing HC11 cells using our transcriptome data published earlier [44]. Finally, to determine which cellular pathways were potentially affected by the selected miRNAs, we conducted a GO and pathway analysis using the differentially regulated target genes identified from the transcriptomics data (figure 1).

### 3.2. Prediction of miRNAs targeting the MMTV genome

**Figure 2.**
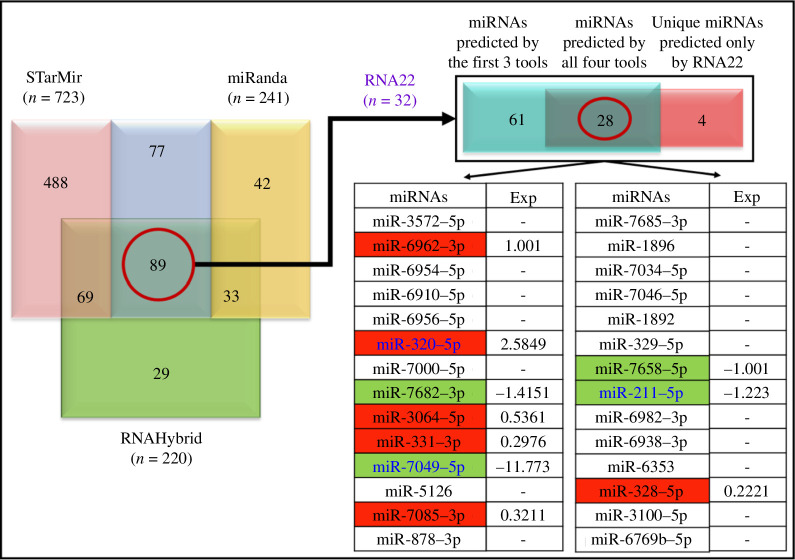
Selection of predicted miRNAs binding to different parts of the MMTV genome. The Venn diagram represents 89 predicted miRNAs using STarMir, miRanda and RNAhybrid that were further screened using the RNA22 tool. This resulted in 28 common miRNAs selected by all four tools, while 61 were predicted by the first three tools only, and four were uniquely predicted by RNA22. The table lists the 28 miRNAs predicted by all four tools. The ones highlighted in red were upregulated, while the ones highlighted in green were downregulated upon MMTV expression in HC11 mammary epithelial cells [45]. The remaining 61 miRNAs were not detected in our miRNAseq data. The three miRNAs highlighted in blue among these were the ones characterized further in this study based on their significant *p* and *q* values (both < 0.05). Dashes indicate miRNAs not detected in our miRNAseq data.

To identify host miRNAs that can target the MMTV genome, we employed four bioinformatics tools that could find MMTV–miRNA target binding sites. Towards this end, sequences from the MMTV C3H strain were used to predict host miRNAs with the capability to bind the MMTV genome using first only three of the following four bioinformatic tools: STarMir, miRanda and RNAhybrid (figures 1 and 2). The tool STarMir conducts an estimation of miRNA binding sites for a variety of species. This tool utilizes high-throughput miRNA−mRNA interaction data from cross-linking immunoprecipitation studies and logistic probability-based prediction models to analyse the data [56,57]. This framework considers sequence, structure and thermodynamic status for seed and seedless miRNA-target duplexes [56]. For secondary structure elucidation, STarMir mainly uses the prediction tool Sfold [56]. Meanwhile, miRanda (http://www.microrna.org/microrna) filters candidate miRNA−mRNA duplexes by estimating their free energy and uses Vienna RNA Fold (http://rna.tbi.univie.ac.at/cgi-bin/RNAWebSuite/RNAfold.cgi) for RNA folding predictions [58]. The RNAhybrid tool (https://bibiserv.cebitec.uni-bielefeld.de/rnahybrid) classifies the hybridization of small RNAs to larger RNAs depending upon their thermodynamic stability and bond accessibility [59]. A lower free-energy value is indicative of stronger bonding; thus, more trustworthy for target predictions [47]. Although a low hybridization energy indicates a well-predicted miRNA−mRNA complex, estimating appropriate free-energy thresholds remains challenging [20,47]. Finally, RNA22 was used to finalize the list of predicted miRNAs, a tool that uses a pattern-based technique to identify binding sites and associated miRNA/mRNA complexes.

It is important to note that most existing methods use dynamic programming, hidden Markov models or statistical analysis to identify miRNA binding sites, relying on algorithms and often considering species conservation. Yet, confirming miRNA binding sites in target genes is hard, and predictions often differ. RNA22 searches for specific sequences or motifs in mRNA targets or viral genomes that resemble known miRNA binding site characteristics, such as their start sequence or unique structural features. Furthermore, it evaluates the free energy of paired target islands with candidate miRNAs. More precisely, it identifies binding sites employing a minimum of 12 paired bases between the miRNA−mRNA duplex, a free-energy threshold of 12 Kcal mol^−1^, a seed size of 7 nt by default, allowance of G:U wobbles in the seed region and a maximum of two unpaired bases in the seed [60–63]. Thus, pattern-based methods offer unique advantages in terms of sensitivity and noise resistance and are valuable as they can discover new miRNAs without relying solely on species conservation [62].

**Figure 3.**
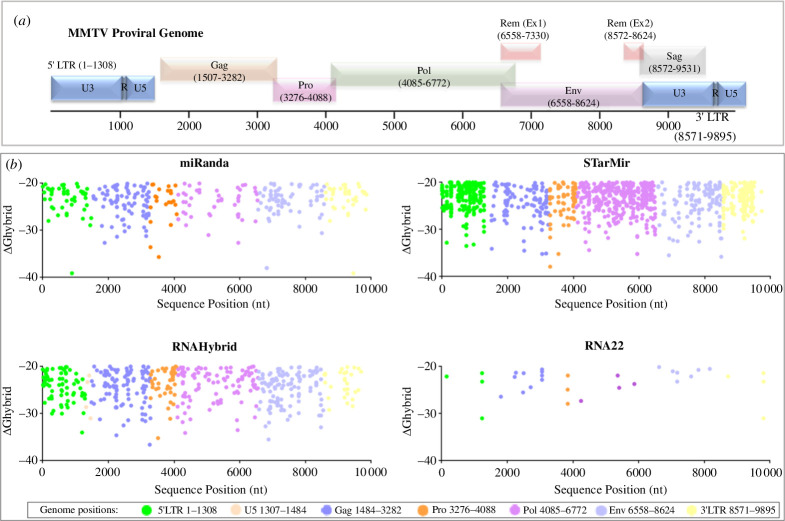
Positions of the predicted miRNAs targeting the MMTV proviral genome. (*a*) Schematic of the MMTV genome with the number line representing the nucleotide number of the C3H strain of MMTV. The R and U5 represent the non-coding regions of the genome. (*b*) Genomic positions of the miRNAs as predicted by the tools miRanda, STarMir, RNAhybrid and RNA22. The coloured dots represent different parts of the MMTV genome, as shown in the key below the graphs, showing minimum hybridization energy or folding energy (ΔGhybrid) against each predicted target. All programs were run at default settings while keeping the MFE value of Δ*G* = −20.0.

Using the prescribed settings, the first three tools (STarMir, miRanda and RNAhybrid) predicted 723, 241 and 220 miRNAs, respectively, that could bind/target different parts of the MMTV genome (figures 2 and 3). Comparative analysis using the Venn diagram exhibited 89 common miRNAs predicted by these three programs (figure 2). The results were further refined using the RNA22 tool, which predicted 32 miRNAs, of which 28 were the same as those predicted by the other three tools, 4 were unique to RNA22 and 61 were predicted by the other three tools only (figure 2; electronic supplementary material, data 1). Figure 3 presents the targeted positions of all predicted miRNAs, showing ΔGhybrid <−20 kcal mol^−1^ against different parts of the MMTV genome. The binding energies are provided in electronic supplementary material, data 1. As can be seen, essentially all parts of the viral genome, both coding and non-coding sequences, were observed to be potential targets of host miRNAs.

### 3.3. Validation of predicted miRNAs using miRNAseq data from MMTV-expressing cells

To determine the biological relevance of the 28 miRNAs identified above for the MMTV life cycle, we generated miRNAseq data from control mouse mammary epithelial HC11 cells and HC11 cells stably expressing HYB MTV [44], a molecular clone of MMTV [45]. Data obtained from the miRNAseq analysis revealed 733 miRNA transcripts dysregulated upon MMTV expression, of which 446 were upregulated and 287 were downregulated. These data were further analysed for differential gene expression to identify those miRNAs that had their expression significantly altered by MMTV (fold change (FC) ≥ 1 or FC ≤ −1). When the 28 predicted and shortlisted miRNAs were compared with our miRNAseq data, we found that out of the 28 predicted miRNAs, the expression of only 10 of them could be detected in MMTV-expressing cells (figure 2). The remaining miRNAs were below the detection threshold. Of these ten, six had an FC > ±1 and four had an FC < ±1 due to MMTV expression (figure 2). The observed differentially regulated miRNAs are highlighted in red and green in figure 2, where red indicates up-regulation and green indicates downregulation after MMTV expression. In light of these results, we decided to delve deeper into the top three miRNAs that showed significant dysregulation upon MMTV expression, as evidenced by considerably lower *p* and *q* values (< 0.05): miR-7049-5p, miR-211-5p and miR-320-5p (highlighted in blue in figure 2). Notably, while miR-320-5p exhibited an increase in expression, miR-7049-5p and miR-211-5p showed a decrease upon MMTV expression.

### 3.4. Prediction of miRNA binding sites on the MMTV genome and their conservation analysis

**Figure 4.**
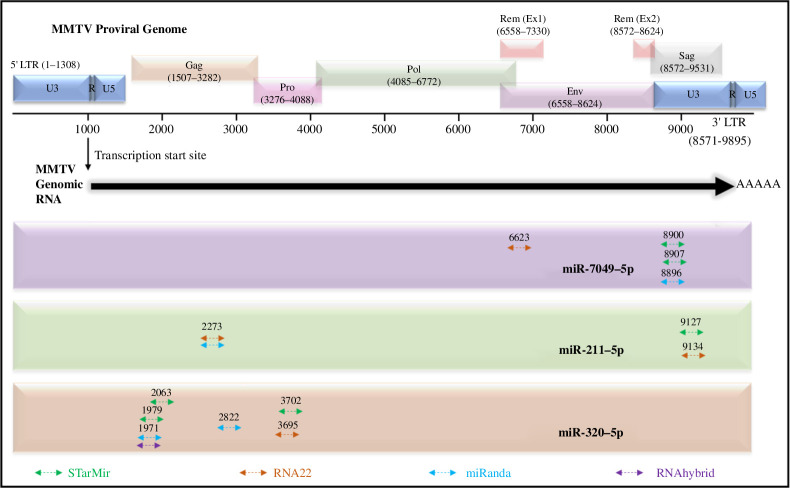
Mapping of the binding sites of the three selected miRNAs on the MMTV proviral genome dysregulated in HC11 cells after MMTV expression identified through miRNAseq**.** The top of the figure shows the MMTV proviral DNA after its integration into the host cells which contains two LTRs flanking the viral genes. The MMTV genomic RNA, shown as a thick black arrow below the proviral genome, initiates from the 5′ repeat, (R) region and ends after the 3′ R region, ending in a stretch of poly As. Thus, it has only one ‘Unique’ region at the 5′ end (U5) and one ‘Unique’ region at the 3′ end (U3). The predicted miRNA binding sites were mapped against the MMTV genomic RNA. The coloured ⟷ lines represent the miRNA binding sites predicted by STarMir, RNA22, miRanda and RNAhybrid. Only the starting position from the 5′ end is mentioned for each binding site. The C3H strain of MMTV (accession no. AF228552.1) was used for the miRNA binding site and conservation analysis (table 1).

Next, we predicted the binding sites of these three selected miRNAs on the MMTV genome. A total of 15 possible binding sites were predicted by the four tools for these miRNAs, several of which were either overlapping or identical. Figure 4 illustrates the target sites of each miRNA on the MMTV genome, while table 1 describes the binding positions and binding energy scores for each of these miRNAs. These three selected miRNAs showed putative binding sites at various locations on the MMTV genome, mainly within the U3/Sag (a region common to all MMTV spliced and genomic RNAs) and some in the Gag and Pro/Pol regions (a region present only in unspliced genomic RNA; figure 4), suggesting that both genomic RNA and spliced mRNAs could be targeted by these miRNAs. The targeted sites included binding to regions starting with nucleotide positions: 6623, 8896, 8900 and 8907 for miR-7049-5p; positions 2273, 9127 and 9134 for miR-211-5p; and positions 1971, 1979, 2063, 2822, 3695 and 3702 for miR-320-5p of the MMTV genome.

**Table 1.** Binding energies and conservation analysis of the miRNA binding sites observed in five different strains of MMTV with the seed sequences of the three selected miRNAs. The miRNA binding sites (MREs) observed in the MMTV genome (designated by nucleotide numbers) are highlighted in bold if fully conserved, in italics if partially conserved (1 change) and in bold italics if not conserved (2 changes), among the five different strains of MMTV tested for the binding and conservation analysis.

description of the miRNA (seed in bold)	miRNAseq fold change *p*‐value	STarMir		RNA22		miRanda		RNAhybrid	
		MMTV position	energy	MMTV position	energy	MMTV position	energy	MMTV position	energy
**miR-7049-5**p **(2 sites)** u**cugaagg**cagucagguaggcgg-23-mer	log_2_ = −11.73 *p* < 0.05	**8900**	−26.80	**6623**	−20.10	**8896**	−25.37		
		**8907**	−26.80						
**miR-211-5p (2 sites)** u**ucccuuu**gucauccuuugccu-22 mer	log_2_ = −1.22 *p* < 0.01	*9127*	−22.10	*2273*	−23.00	*2273*	−27.05		
		*9134*	−22.10						
**miR-320-5p (4 sites)** g**ccuucuc**uucccgguucuucc-22 mer	log_2_ = 2.58 *p* < 0.001	**3702**	−29.10	**3695**	−24.01	***2822***	−22.40	**1971**	−26.80
		**1979**	−27.01			**1971**	−22.40		
		**2063**	−27.01						

As can be seen, several sites were overlapping, such as the sites starting with nucleotides 8896, 8900 and 8907 that probably fell within the same site. The similarities in the binding energies belonging to these overlapping sites support this assertion (table 1). For example, the binding energies of nt positions 8900 and 8907 were identical (−26.80) and very similar to that of position 8896 (−25.37), even though two different tools predicted these energies, while the binding energy for the sites at positions 9127 and 9134 was also identical at −22.10 (table 1). Considering all overlapping and identical sites, the predicted sites were reduced to only eight with the sites represented by 8896/8900/8907 and the nt positions 9127/9134 representing two individual sites in genomic RNA. Therefore, a total of two sites were predicted for miR-7049-5p (nt 8896/8900/8907 and nt 6623), two for miR-211-5p (nt 2273 and nt 9127/9134) and four for miR-320-5p (nt 1971/1979, nt 2063, nt 2822 and nt 3695/3702; see figure 4 and table 1).

Next, we conducted a conservation analysis of the eight miRNA binding sites (i.e. MREs) predicted within the MMTV genome, by using whole-genome sequences from five different strains of MMTVs. The two MREs of miR-7049-5p at nt 8896/8900/8907 and 6623 were observed to be fully conserved (as shown in bold in table 1), despite changes of 5- and 2-nucleotides in the rest of the non-seed-binding sequence, respectively. On the other hand, the two predicted MREs of miR-211-5p at nt 2273 and nt 9127/9134 showed one unique variation and thus were considered partially conserved, as shown in italics in table 1. The rest of the non-seed sequence was conserved around the site at nt 2273 but showed two variations around the site at 9127/9134. Finally, of the four MREs analysed for miR-320-5p, the binding site at nt 2822 was the least conserved among the eight binding sites analysed, showing two differences within the 7-nucleotide binding site and two differences outside the non-seed binding region (shown in bold italics in table 1). The other three MREs (nt 1971/1979, nt 2063 and nt 3695/3702) for this miRNA were fully conserved within the different strains of MMTV (shown in bold in table 1), while only the site at nt 2063 showed a 2-nucleotide variation outside the binding site.

Taken together, this analysis suggests that miR-7049-5p and miR-320-5p have the potential to target all the tested strains of MMTV using the conserved viral MREs, while the more variable MRE of miR-211-5p suggests that miR-211-5p may be more efficient at targeting some, but not other strains of MMTV. This assertion needs to be tested empirically for further validation.

### 3.5. miRNA−mRNA target site docking

**Figure 5.**
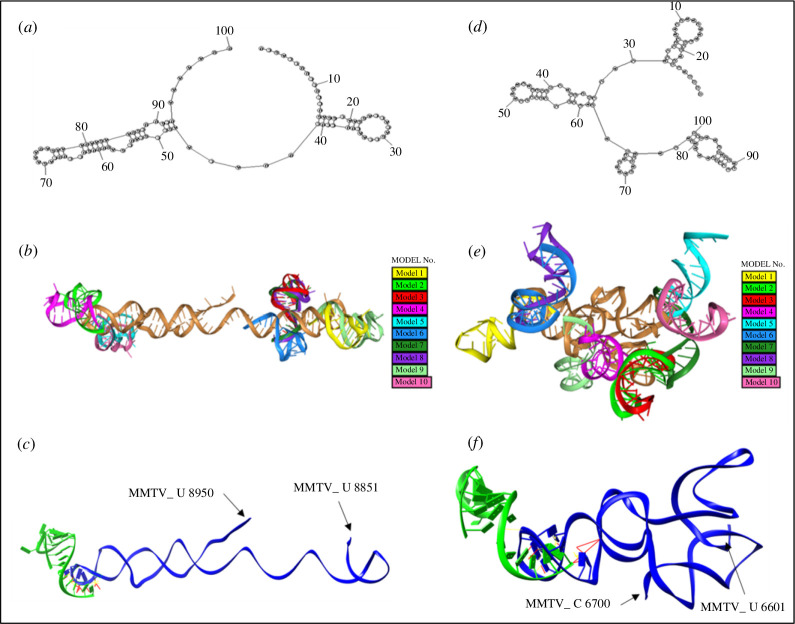
Docking complexes of miR-7049-5p with the MMTV mRNA at the predicted positions. (*a–c*) Docking complex of miR-7049-5p with MMTV mRNA in the vicinity of nucleotide position 8896/8900/8907. MMTV mRNA sequence (U 8851–U 8950 nt) was docked with miR-7045-5p. (*a*) Representation of the 2D structure of the corresponding MMTV mRNA sequence. (*b*) The top 10 predicted 3D miRNA–mRNA docking configurations are displayed. The MMTV mRNA is depicted as a gold ribbon, while various colourful structures represent miR-7049-5p docking at distinct positions on the same mRNA molecule near position 8896/8900/8907. (*c*) The best predicted model no. 2 (depicted as green ribbon) reveals hydrogen bonding and contact points (CPs) with MMTV mRNA (depicted as blue ribbon). (*d–f*) Docking complex of miR-7049-5p with MMTV mRNA in the vicinity of nucleotide position 6623. MMTV mRNA sequence (U 6601–C 6700 nt) was docked with the miR-7045-5p. (*d*) Representation of the 2D structure of the designated mRNA sequence. (*e*) The top 10 anticipated 3D miRNA–mRNA docking configurations are displayed. The mRNA is depicted as a gold ribbon, while various colourful structures represent miR-7049-5p docking at distinct positions on the same mRNA molecule near position 6623. (*f*) The best predicted model no. 1 (depicted as green ribbon) reveals hydrogen bonding and CPs with MMTV mRNA (depicted as blue ribbon).

**Figure 6.**
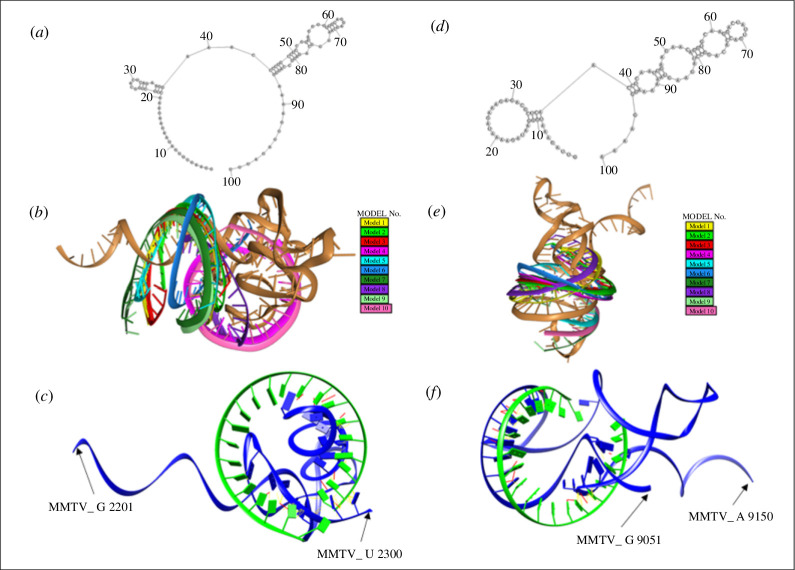
Docking complexes of miR-211-5p with MMTV mRNA at the predicted positions. (*a–c*) Docking complexes of miR-211-5p with MMTV mRNA in the vicinity of nucleotide position 2273. MMTV mRNA sequence (G 2201–U 2300 nt) was docked with miR-211-5p. (*a*) Representation of the 2D structure of the corresponding MMTV mRNA sequence. (*b*) The top 10 anticipated miRNA–mRNA docking configurations in 3D are displayed. The mRNA is depicted as a gold ribbon, while various colourful structures represent miR-211-5p docking at distinct positions on the same mRNA molecule near nucleotide position 2273. (*c*) The best predicted model no. 4 (depicted as green ribbon) reveals hydrogen bonding and CPs with MMTV mRNA (depicted as blue ribbon). (*d–f*) Docking complex of miR-211-5p with MMTV mRNA in the vicinity of nucleotides 9127/9134. MMTV mRNA sequence (G 9051–A 9150 nt) was docked with the miR-211-5p. (*d*) Representation of the 2D structure of the designated mRNA sequence. (*e*) The top 10 anticipated miRNA–mRNA docking configurations in 3D are displayed. The mRNA is depicted as a gold ribbon, while various colourful structures represent miR-211-5p docking at distinct positions on the same mRNA molecule near position 9127/9134. (*f*) The best predicted model no. 1 (depicted as green ribbon) reveals hydrogen bonding and CPs with MMTV mRNA (depicted as blue ribbon).

**Figure 7.**
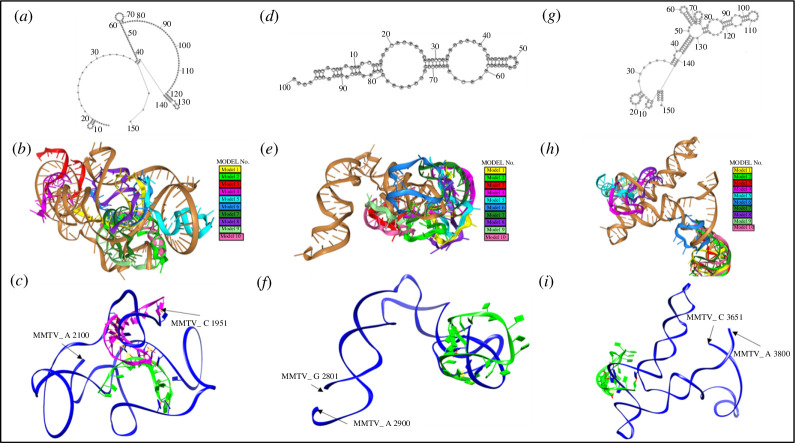
Docking complexes of miR-320-5p with MMTV mRNA at the predicted nucleotide positions. (*a–c*) Docking complexes of miR-320-5p with MMTV mRNA in the vicinity of nucleotide positions 1971/1979 and 2063. MMTV mRNA sequence (C 1951–A 2100 nt) was docked with the miR-320-5p. (*a*) Representation of the 2D structure of the selected MMTV mRNA sequence. (*b*) The top 10 anticipated miRNA–mRNA docking configurations in 3D are displayed. The mRNA is depicted as a gold ribbon, while various colourful structures represent miR-320-5p docking at distinct positions on the same mRNA molecule near positions 1971/1979 and 2063. (*c*) The best predicted models no. 9 (depicted as green ribbon) and no. 6 (depicted as magenta ribbon) reveal hydrogen bonding and CPs with MMTV mRNA near position 1971/1979 and 2063, respectively. (*d–f*) Docking complex of miR-320-5p with MMTV mRNA in the vicinity of nucleotide position 2822. MMTV mRNA sequence (G 2801–A 2900 nt) was docked with the miR-320-5p. (*d*) Representation of the 2D structure of the corresponding MMTV mRNA sequence. (*e*) The top 10 anticipated miRNA–mRNA docking configurations in 3D are displayed. The mRNA is depicted as a gold ribbon, while various colourful structures represent miR-320-5p docking at distinct positions on the same mRNA molecule near nucleotide position 2822. (*f*) The best predicted model no. 6 (depicted as green ribbon) reveals hydrogen bonding and CPs with MMTV mRNA near nucleotide position 2822. (*g–i*) Docking complex of miR-320-5p with MMTV mRNA in the vicinity of nucleotide position 3695/3702. MMTV mRNA (C 3651–A 3800 nt) was docked with the miR-320-5p. (*g*) Representation of the 2D structure of the designated MMTV mRNA sequence. (*h*) The top 10 anticipated miRNA–mRNA docking configurations in 3D are displayed. The mRNA is depicted as a gold ribbon, while various colourful structures represent miR-320-5p docking at distinct positions on the same mRNA molecule near nucleotide position 3695/3702. (*i*) The best predicted model no. 9 (depicted as green ribbon) reveals hydrogen bonding and CPs with MMTV mRNA (depicted as blue ribbon).

The next step was to dock the three identified miRNAs at the predicted binding sites of the MMTV genome for further confirmation and to determine the binding potential of the biomolecules (figures 5–7). To dock miRNAs onto predicted MMTV binding regions, we initially generated 2D structures of both the miRNAs and the MMTV genome. Subsequently, these 2D structures (along with the nucleotide sequences of the miRNAs and MMTV genome) were utilized to create the corresponding 3D structures. However, due to size limitations of the available software (which typically allows for sequences of up to 500 nucleotides), we addressed this challenge by focusing on sequences (about 100–150 nt long) in close proximity to the predicted miRNA binding sites. We used the HNADOCK server for docking the 3D structures of both the miRNAs and the MMTV genome. This server accommodates both pre-modelled 3D structures and simple nucleotide sequences of mRNAs. For RNA sequences, the server utilizes the HMODELRNA protocol to search for adjacent templates from a database or employs the 3dRNA protocol for *ab initio* modelling and prediction in cases where no template is available. The server generated 10 or more refined structures that were subsequently assessed and visualized using UCSF Chimera [52].

It is worth noting that for RNA, the secondary structure describes how nucleotides base pair to form local folding patterns, including features like stem-loop structures, bulges and internal loops. Computational algorithms predict this structure by analysing the sequence and base-pairing interactions, based on thermodynamic principles that are typically represented graphically. On the other hand, the 3D structures of RNAs refer to their spatial arrangement, including the overall folding of the molecule, as well as interactions between distant regions of the RNA sequence. These are determined through experimental techniques like X-ray crystallography, nuclear magnetic resonance spectroscopy and cryo-electron microscopy. When the 3D structure aligns with the predicted secondary structure, it suggests that the observed 3D arrangement of the RNA molecule matches the predicted base-pairing interactions and secondary structure features, confirming the accuracy of computational predictions, and increasing our understanding of how RNA structure impacts its function [64–67].

In our study, for all predicted mRNAs, their 3D structures were highly compatible with their predicted secondary RNA structures, suggesting that the predictive algorithms used accurately reflected the actual folding of the RNA molecule in its native environment (see the example discussed below). Additionally, for most of the binding positions that were close to each other (e.g. positions 8896/8900/8907), the same mRNA template was used for docking. Briefly, the HNADOCK bioinformatic tool predicted 100 possible interaction structures based on the lowest binding energy, known as the docking score, and the top 10 docked results were further analysed for each miRNA−mRNA complex; the lower the binding energy, the more stable the interaction between the two molecules. Similarly, the software provided a root mean square deviation (rmsd) value, providing an estimate of the degree of structural similarity between two molecules after optimal superposition of their 3D structures.

### 3.6. Docking of miR-7049-5p with the MMTV genome

The predicted 3D structures of both miR-7049-5p and MMTV mRNA were docked to analyse the binding potential and nucleotide–nucleotide interactions at all predicted binding sites (figure 5). As shown in figure 4, miR-7049-5p exhibited two potential highly conserved MREs on the MMTV genome, the first one starting from nt position 6623, predicted by RNA22, while the second one starting from nt 8896/8900/8907, predicted by both STarMir and miRanda. These sites were further assessed for miRNA–mRNA interactions through 3D structure docking analysis. This analysis revealed that miR-7049-5p could potentially bind to these MMTV genomic positions through various 3D configurations and interactions. Figure 5 visually demonstrates the miRNA–mRNA binding conformations to the predicted binding sites.

Figure 5*a* shows the 2D structure of approximately 100 nt RNA sequence from the MMTV genome used to dock miR-7049. Figure 5*b*, on the other hand, shows the 3D structure of this region as a gold ribbon containing the MRE that starts with nucleotide 8896/8900/8907 along with the top 10 predicted interactions of this region with miR-7049, as shown in ribbons of various colours randomly assigned by the program. For this site, the docking model no. 2 demonstrated the most favourable conformation that was based on the ability of the two molecules (the miRNA and the MRE on the MMTV genome) to form hydrogen (H) bonds and maintain contact points (CPs) with each other (figure 5*c*). This model demonstrated 17 hydrogen bonds and 32 CPs between the miRNA and the MMTV mRNA in this region (figure 5*b*,*c*; electronic supplementary material, data 2 and 3). Electronic supplementary material, data 2 file contains higher resolution images of the top 10 models with relevant data, while electronic supplementary material, data 3 file provides the raw data of the hydrogen bonds and CPs between the two molecules. The two stem-loops observed in the 2D structure of the MRE in figure 5*a* were clearly visible as two α-helices in figure 5*c* when the remaining miRNA binding interactions were removed from the figure, with the bigger stem-loop between nt 45 and nt 95 having a longer α-helical structure than the one observed between nt 15 and nt 41. This served to confirm our earlier assertion that we observed good concordance between our 2D and 3D predicted structures.

Similarly, figure 5*d* shows the 2D structure of approximately 100 nt mRNA sequence from the MMTV genome containing the MRE at position 6623 used to dock miR7049. As observed, this structure showed four separate stem-loops. For this site, the 3D model no. 1 emerged as a potentially good binding configuration, demonstrating 12 hydrogen bonds and 31 CPs between the miRNA and the MMTV mRNA in this region (figure 5*e*,*f*; electronic supplementary material, data 2 and 3). Some of the other models revealed plenty of CPs, but no actual hydrogen bonding, such as model no. 6 for nt position 6623 which showed 33 CPs, but no hydrogen bonds (electronic supplementary material, data 3). Such models were removed from further consideration since complementary hydrogen bonding between the seed and MRE is a prerequisite for the ability of the miRNA to target the relevant mRNAs. These docking analyses suggest specific binding interactions between miR-7049-5p and the MMTV genome at both the indicated positions with the MRE at position 8896/8900/8907 having a stronger binding potential for miR-7049-5p than the position at nt 6623, based on the number of hydrogen bonds and CPs.

### 3.7. Docking of miR-211-5p with the MMTV genome

Target prediction analysis for miR-211-5p also suggested two potential binding sites in the MMTV genome, starting from nt 2273 and nt 9127/9134 (figure 4 and table 1). For the position at nt 2273, predicted by both RNA22 and miRanda, the docking analysis identified model no. 4 as the optimal binding configuration among the ten models (figure 6*b*,*c*). This model exhibited 20 hydrogen bonds and 92 CPs between the miRNA and mRNA (electronic supplementary material, data 2 and 3). Similarly, docking miR-211-5p with the MMTV genome at nt position 9127/9134 revealed model no. 1 as the most favourable interaction. This model showed 26 hydrogen bonds and 52 CPs (figure 6*e*,*f*; electronic supplementary material, data 2 and 3). Similar to model no. 6 of nt position 6623 for miR-7049-5p, model no. 8 of miR-211-5p showed plenty of CPs (*n* = 115), but no hydrogen bonding, leading to its exclusion as a possible site of interaction (electronic supplementary material, data 3). These findings suggest that miR-211-5p can potentially target the MMTV genome at both positions effectively, given the high number of hydrogen bondings observed.

### 3.8. Docking of miR-320-5p with the MMTV genome

The miR-320-5p exhibited binding potential at four distinct positions on the MMTV genome, starting from nucleotide positions 1971/1979, 2063, 2822 and 3695/3702 (figure 4 and table 1). While position 2063 and position 2822 were predicted by STarMir and miRanda only, respectively, position 3695/3702 was predicted by STarMir and RNA22, and position 1971/1979 was predicted by three tools (STarMir, miRanda and RNAhybrid). Docking models no. 9 (shown in green in figure 7*c*) and no. 6 (shown in magenta in figure 7*c*) demonstrated the optimal alignments near the mRNA positions 1971/1979 and 2063. Due to the close proximity of these sites, they were modelled using the same region of the MMTV genomic RNA (nt 1951–2100). We could observe 25 hydrogen bonds and 59 CPs for model no. 9, while model no. 6 showed nine hydrogen bonds and 30 CPs, suggesting that both these sites at positions 1971/1979 and 2063 could serve as potential miRNA target sites (figure 7*b*,*c*; electronic supplementary material, data 2 and 3). Interestingly, nt 1971 was the only binding site predicted by RNAhybrid for the three validated miRNAs (table 1). For the mRNA position at nt 2822, model no. 6 emerged as the best candidate, forming 12 hydrogen bonds and 22 CPs (figure 7*e*,*f*; electronic supplementary material, data 2 and 3). Similarly, for the mRNA sequence at positions 3695/3702, the docking model no. 9 exhibited the most suitable interactions (figure 7*h*,*i*; electronic supplementary material, data 2 and 3), forming 12 hydrogen bonds and eight CPs. These findings show that miR-320-5p has the potential to effectively target the MMTV genome at the four positions interrogated, with position 1971/1979 being the most favourable one, based on the strong 25-hydrogen bond interaction observed.

Altogether, the docking analysis supports the assertion that host miRNAs have the potential to target the MMTV genome, thereby regulating MMTV infection and replication.

### 3.9. The RNAi machinery targets the MMTV genome

**Figure 8.**
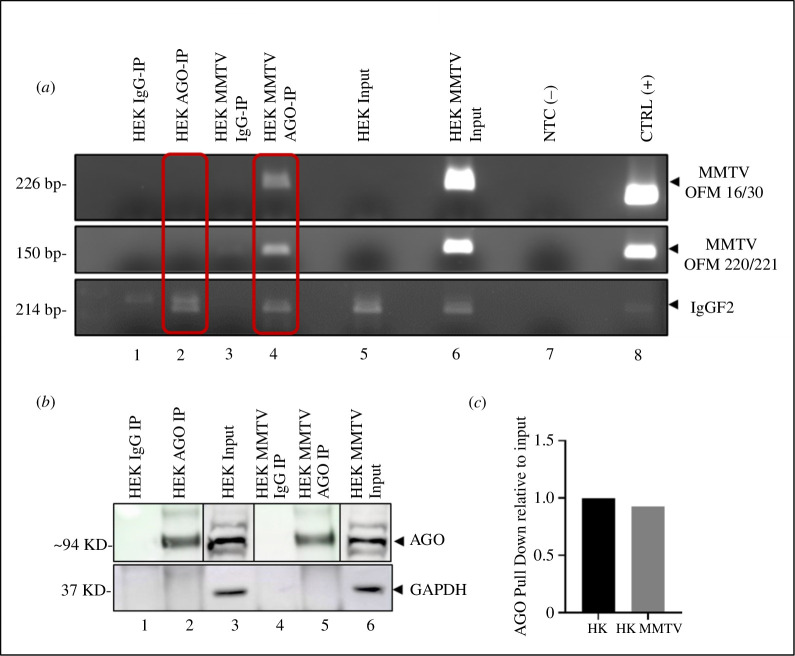
The RNAi machinery targets the MMTV genome**.** RIP analysis of MMTV RNAs associated with AGO proteins using control anti-IgG and anti-pan AGO antibodies that detect all forms of AGOs in the cells. (*a*) RT-PCR analysis of anti-IgG or anti-AGO-RNA immunoprecipitated RNAs using two MMTV-specific primer sets (OFM16/OFM30 and OFM220/OFM221). The red boxes highlight the results of the RT-PCR analysis from cells with and without MMTV. IGF2 was used as the AGO-RNA IP internal control. NTC (−) indicates no template negative control. CTRL(+) represents a cDNA sample from an MMTV-induced tumour cell line (MM5MT) expressing high levels of MMTV. (*b*) Western blot analysis of proteins from the immunoprecipitated samples and the input lysates using an anti-pan AGO antibody as a control for RNA IP pull-down. GAPDH was used as an endogenous control in the Western blot. (*c*) Densitometric analysis of the Western blot shown in (*b*). Densitometry was performed using GelQuant software, and immunoprecipitated AGO protein levels were represented relative to the AGO signal from the input.

The *in silico* investigations of the potential of the cellular host miRNAs to target the MMTV genome led us next to empirically test whether one of the primary effectors of miRNA biogenesis, AGO, could interact with the MMTV genome. This was accomplished by conducting an RNA ‘pull-down’ experiment using an anti-AGO antibody. As mentioned earlier, AGO plays a central role in RNAi by directly binding to the active miRNA/mRNA complex as part of RISC [6–10]. Thus, cell lysates from a control (HEK) or an MMTV-expressing stable cell line (HEK-MMTV) established previously [45] were incubated with either a control isotype-specific IgG or anti-AGO antibody conjugated to magnetic beads. The beads were separated to ‘pull-down’ RNA interacting with AGO. Finally, the RNA eluted from the beads was subjected to RT-PCR after DNase treatment to eliminate any contaminating DNA. cDNAs were prepared to detect MMTV RNA using two different primer sets targeted to the viral genome (figure 8*a*; electronic supplementary material, data 4). This way, we could evaluate the potential immunoprecipitation of MMTV RNA by the anti-AGO antibody in comparison with control cells not expressing MMTV.

As expected, amplification of cDNAs prepared from immunoprecipitated RNAs from HEK cells (not expressing MMTV) using isotype-specific control or AGO antibodies revealed a lack of MMTV-specific bands from either set of primers (lanes 1 and 2; figure 8*a*). This was despite the fact that the expected sized bands could be observed in lanes 6 and 8 displaying the input sample, not immunoprecipitated with anti-AGO antibody, from HEK-MMTV cells and the positive control for PCR (CTRL(+)) representing a cDNA sample prepared from an MMTV-induced tumour cell line expressing high levels of the virus, respectively. This was followed by the RT-PCR analysis of test cDNAs prepared from immunoprecipitated RNAs isolated from HEK-MMTV cells using the same isotype-specific control and AGO antibodies (lanes 3 and 4; electronic supplementary material, data 4). As demonstrated in lanes 2 and 4 highlighted in red boxes, the presence of a band of the right size in the HEK-MMTV AGO-IP lane as opposed to the control HEK AGO-IP lane with both sets of primers verified the presence of MMTV RNA that co-immunoprecipitated with the anti-AGO antibody (lane 4), but not the isotype control antibody (lane 3). IGF2 served as the internal RNA IP control to confirm that each sample had amplifiable cDNAs.

To ensure that our pan-AGO antibody that was used for the immunoprecipitation could detect AGO proteins successfully, input and immunoprecipitated samples from HEK and HEK-MMTV cells were further analysed with the same pan-AGO antibody in a Western blot. As can be seen in figure 8*b* and electronic supplementary material, data 4, the presence of the right-sized band in lanes 2, 3, 5 and 6 containing input and immunoprecipitated samples from anti-AGO antibody and its absence in lanes 1 and 4 containing immunoprecipitated samples from the control antibody confirmed that the pan-AGO antibody used was specific and able to recognize AGO proteins of the right size in our samples. Furthermore, densitometric analysis was carried out for the band intensities of AGO in figure 8*c*, lanes 3 and 6), and intensities of immunoprecipitated protein levels were plotted with the corresponding input values. As can be seen, the amount of AGO proteins expressed in the two cell lines was comparable, ensuring that the absence of bands in HEK was not due to low levels of AGO proteins (figure 8*c*). Together, these results confirm a direct physical interaction of MMTV RNA with AGO, a critical enzyme of RISC involved in facilitating the targeting of cellular mRNAs by miRNAs, supporting our assertion that the MMTV genome can be targeted by host miRNAs.

### 3.10. Target gene predictions for the selected miRNAs within the host genome

**Figure 9.**
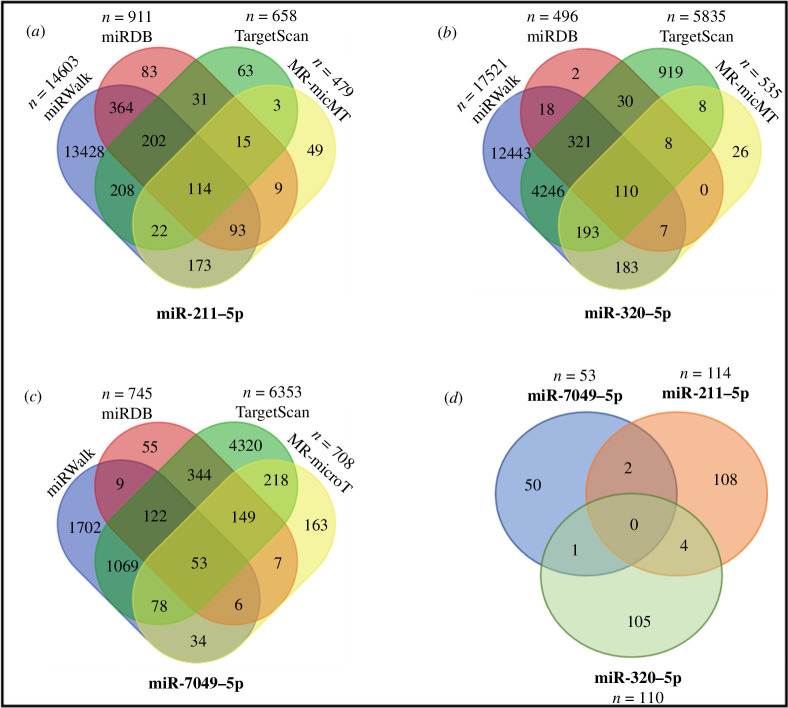
Target gene prediction of miR-211-5p, miR-320-5p and miR-7049-5p**.** Targeted genes of the indicated miRNAs were predicted using four online tools, including miRWalk, miRDB, TargetScan and MR-microT. Venn diagram of the target genes predicted for (*a*) miR-211-5p, (*b*) miR-320-5p and (*c*) miR-7049-5p. (*d*) Venn diagram showing common predicted genes for each miRNA.

Now that we demonstrated that the MMTV genome can be targeted by host miRNAs and identified three miRNAs with this potential that were differentially regulated by MMTV, we next were interested in the cellular genes targeted by the same three miRNAs. This was especially important since the targeted genes could be critical in understanding how the host cell mounts a response to the viral infection and how the virus may circumvent such antiviral responses to facilitate its own replication, persistence and survival. Therefore, we used various online miRNA-target prediction servers, including miRWalk, miRDB, TargetScan and MR-microT, to predict the potential host target genes of the identified miRNAs. Figure 9 illustrates Venn diagrams that indicate the number of predicted target genes identified by each program. Specifically, 114, 110 and 53 genes were commonly targeted by miR-211-5p, miR-320-5p and miR-7049-5p, respectively, across all four prediction tools (figure 9*a*–c). Figure 9*d* shows Venn diagrams of the number of genes that were commonly targeted by each of the three miRNAs. As can be seen, the cellular targets of each miRNA were quite specific since only a few gene targets were shared between them: only one between miR-7049-5p and miR-320-5p, two between miR-7049-5p and miR-211-5p and four between miR-211-5p and miR-320-5p. Interestingly, no common predicted target gene was shared among the three miRNAs (figure 9*d*). These observations suggest that each of these miRNAs (whose expression was affected by MMTV and who can potentially target the MMTV genome) could target a different set of host genes, exhibiting a unique potential to affect the host. This diversity should enhance the range of cellular pathways influenced by their induction and how they affect virus infection.

### 3.11. Validation and functional annotation of the predicted target genes

Subsequently, we analysed the expression of the cellular genes targeted by the three miRNAs to determine if MMTV affected their expression. This was achieved by using our mRNAseq data published earlier [44] from the same cells from which we obtained our miRNAseq data presented in figure 2 in this study. Almost every predicted gene for each miRNA was present in our mRNAseq data; however, table 2 shows only those having more than ±2-fold (*q* < 0.05) dysregulation upon MMTV expression. In particular, a significant proportion of these genes exhibited downregulation by the corresponding miRNAs: 61.5% (8/13) for miR-211-5p; 75% (9/12) for miR-320-5p; and 87.5% (7/8) for miR-7049-5p, resulting in an average inhibition of 75%. Interestingly, this pattern closely mirrors our earlier findings, where we observed an overall 74% inhibition of cellular genes differentially regulated by MMTV [44]. This suggests that the differentially regulated miRNAs identified in this study may be involved in some of the inhibition being observed at the host gene level after MMTV infection, an assertion that needs to be verified experimentally.

**Table 2.** Validation of target genes for each of the three selected miRNAs. Only genes showing more than ±2-fold dysregulation upon MMTV expression are listed using previously published mRNAseq data [44]. Italics indicate upregulated genes; bold indicates downregulated genes.

miR-211-5p		miR-320-5p		miR-7049-5p	
gene symbol	HC11-MMTV versus HC11	gene symbol	HC11-MMTV versus HC11	gene symbol	HC11-MMTV versus HC11
Clmp	*4.134*	Kcnb1	*3.974*	Epha7	*2.676*
Epha7	*2.676*	Prdm14	*2.542*	Diras2	**−4.697**
H2-M2	*2.865*	Tacr1	*2.974*	Lypd6	**−4.660**
Lrp2	*4.037*	Add2	**−3.234**	Mcf2l	**−3.391**
Nostrin	*3.973*	Bcat1	**−5.016**	Negr1	**−3.820**
Fbn2	**−3.052**	Ces2f	**−2.827**	Plin2	**−3.096**
Gxylt2	**−5.154**	G0s2	**−3.220**	Tnmd	**−3.827**
Hapln1	**−3.091**	Grb10	**−2.580**	Unc80	**−2.076**
Lhfpl4	**−2.631**	Irf4	**−2.044**		
Nhsl2	**−5.585**	Nhsl2	**−5.585**		
Ociad2	**−2.511**	Plin2	**−3.096**		
Plin2	**−3.096**	Vipr1	**−5.062**		
Rps6ka5	**−2.112**				

**Figure 10.**
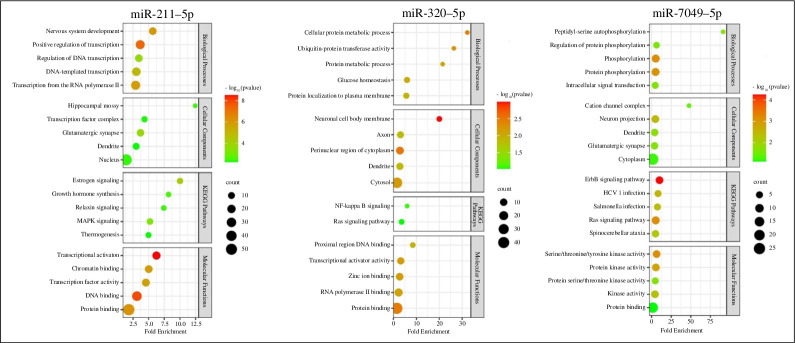
GO analysis of the three identified cellular miRNAs that putatively target the MMTV genome. The cellular pathways impacted by the three miRNAs shortlisted in this study are shown. The heat map shows the statistical significance (*p* values) observed, while the size of the circle in each plot reflects the number of genes affected in each pathway (depicted in black).

To get a better assessment of the cellular pathways affected by the genes targeted by the three miRNAs, we conducted a GO analysis of the confirmed targets of the three interrogated miRNAs through DAVID. This analysis revealed the participation of the three miRNAs in various cellular processes pertinent to MMTV biology, which could impact overall cellular function after MMTV infection (figure 10). These processes encompass pathways associated with hormone and growth factor signalling, gene transcription, nuclear factor kappa B (NF-ƙB), mitogen-activated protein (MAP), rat sarcoma (Ras) and erythroblastic oncogene B (ErbB) signalling, cell metabolism, as well as serine, threonine and tyrosine kinase activity. These pathways are crucial for MMTV replication, aligning with our previous observations regarding the impact of MMTV expression on these pathways [44].

## 4. Discussion

The present study aimed to elucidate the intricate interplay between the host RNA silencing machinery and MMTV through the involvement of miRNAs. We predicted the putative binding sites of host miRNAs on the MMTV genome to determine if it could be targeted directly by cellular miRNAs using several bioinformatics tools (figure 1). Our results revealed that the MMTV genome could be targeted by an array of miRNAs, the binding sites for which were distributed across the genome (figures 2 and 3). Subsequently, a set of rigorous criteria was established, including the MFE threshold of ΔGhybrid < −20 kcal mol^−1^, in order to enhance the precision of our predictions. A total of 89 miRNAs were among the miRNAs predicted by the RNAhybrid, miRanda and STarMir approaches, as determined through a comparative analysis (figure 2). These miRNAs were subjected to additional screening using RNA22 software (figure 2), resulting in a reduction in the initial number of miRNAs to 28, which overlapped with the miRNAs predicted in the previous analysis. These miRNAs were subjected to further screening using our differential miRNAseq data from MMTV-expressing cells (figure 2). This led to successful identification of three miRNAs, miR-7049-5p, miR-211-5p and miR-320-5p, that exhibited differential regulation upon MMTV expression in the mouse mammary epithelial cells (figure 2 and table 1), cells that are the main targets of MMTV replication and tumorigenesis [2].

Consequently, the aforementioned miRNAs were used to predict their binding sites on the MMTV genome using four different tools (figure 3). This revealed the presence of multiple binding sites within regions found exclusively within the gRNA, such as those targeting *gag* and *pro* genes, or U3, regions found in all viral RNAs, both spliced and unspliced (figure 4). Of the three miRNAs analysed, miR-320-5p has the potential to target gRNA only using four different binding sites on the virus, a feature that may allow a broader ability of this miRNA to target different strains of MMTV. It is important to note that the gRNA in retroviruses also serves as the mRNA that codes for the structural and enzymatic proteins of the virus, including the Gag, Pro and Pol. Thus, any miRNA that has the potential to target gRNA can not only modulate progeny virion production but also expression of viral proteins that may have other roles in the viral life cycle, such as immune modulation and cell transformation. Compared to miR-320-5p, miR-7049-5p and miR-211-5p seem more versatile with the potential to target not only gRNA but all the other spliced mRNAs of MMTV, including Env, Rem and Sag. Interestingly, miR-7049 revealed one binding site (starting with nt 6623) that could specifically target the Env and Rem mRNAs of MMTV only, excluding Sag (in addition to the gRNA, of course; figure 4). These two proteins are crucial for MMTV replication and pathogenesis with Rem being important for the nucleocytoplasmic transport of unspliced RNA, while Env being important for not only virus entry into target cells and immune activation via toll-like receptor 4, but also having the ability to transform mammary epithelial cells *in vitro* and influence mammary tumorigenesis in mice [2,68,69].

The miRNA–mRNA target site docking analysis further validated the reliability of our prediction data and identified the most stable binding sites, thereby reinforcing the accuracy of our predictions (figures 5–7). Following the *in silico* analysis showing that host miRNAs could target multiple regions of the MMTV genome and stably interact via numerous hydrogen bonds, the AGO-RNA IP experiment verified that the MMTV genome could actually interact with the AGO protein of RISC (figure 8), confirming the physical interaction between MMTV and host miRNAs. Subsequently, target prediction was conducted for the select miRNAs to understand which host mRNAs they could target, other than the viral RNA, utilizing various online miRNA-target prediction software tools (figure 9 and table 2). It is intriguing to note that the three miRNAs targeted their own particular set of host genes with few overlaps and, in fact, did not exhibit any overlap between the three of them, suggesting that each miRNA could target a different set of cellular pathways (figure 9*d*). Furthermore, analysis of the host genes targeted by these miRNAs revealed an overall inhibition of 75% of those genes, similar to what we have observed in cells expressing MMTV [44]. Finally, KEGG and GO pathway analysis revealed that the predicted miRNAs primarily targeted genes associated with pathways related to Ras signalling, RNA pol II transcription, mitogen-activated protein kinase (MAPK) signalling and others (figure 10), pathways that are critical for virus replication and pathogenesis, as discussed previously [44].

miRNAs, as key effectors of the RNAi machinery, are known to interact with viral genomes and transcripts, as observed in the case of HIV, HCV and hepatitis E virus (HEV), BVDV and others [35–37,70,71]. The host miRNAs that target the viral RNA can behave as proviral or antiviral agents. For example, several host ‘anti-HIV’ miRNAs have been described, including miR-28, miR-223, miR-150 and miR-382, that specifically target the 3′ end of the HIV mRNAs [72,73]. These miRNAs are enriched in both resting CD4^+^ T cells and monocytes, thus keeping the virus expression low, contributing to HIV-1 latency in these cell types compared to activated CD4^+^ T cells or macrophages. These findings suggest that cellular miRNA expression can be modulated to act as part of the host innate immunity to inhibit virus replication in naive cells to establish viral latency, while later on, the expression of these antiviral miRNAs can be suppressed to facilitate the spread of the virus in the host, once these cells are activated (CD4^+^ T cells) or differentiated (macrophages). In contrast, the host miR-122 in hepatoma cells can directly target the HEV genome via binding to a conserved site, leading to enhancement of virus replication, and providing a good target for anti-sense-based antiviral therapy against HEV [74].

In our scenario, we suggest that the presence of MMTV induces specific host miRNAs that target the MMTV genome via the AGO proteins within RISC where the actual miRNA–mRNA targeting takes place. Furthermore, it is now known that the fate of mRNAs targeted by RISC is generally defined inside cytoplasmic RNA–protein complexes/granules (e.g. RNA processing bodies (P-bodies) or stress granules), which prevent the association of these mRNAs from the cellular translational machinery and decide whether the targeted RNAs are degraded or stored for future use [75–77]. It is also well-known that many DNA and RNA viruses induce these mRNA/protein granules, as a ‘stress’ response, such as rotavirus, respiratory syncytial virus, Sindbis, cytomegalovirus, HCV and Rift Valley fever virus. [78–83]. While the primary function of these RNA granules is to mount an antiviral response as part of the host innate immunity, the viruses try to block the formation of these stress granules or disrupt P-body formation using virally encoded factors to facilitate their replication [84]. Thus, depending upon the miRNAs that target the viral genome and which proteins of the RNAi machinery MMTV transcripts can interact with, the fate of the MMTV replication can change.

Not much is known about the normal role of miR-7095 in cellular functions or during viral infections. It has been implicated in apoptosis of neuronal cells as a predicted target of a conserved long non-coding RNA [85]. It has also been reported to be downregulated by Varinostat, a pan-histone deacetylase inhibitor used to reduce vascular inflammation [86]. On the other hand, miR-211 is a well-known tumour suppressor miRNA in triple-negative breast cancer and hepatocellular carcinoma [87–89] and has a strong neuroprotective function [90,91]. However, not much is known about its interaction with viruses. The non-canonical, Drosha-independent miR-320 has been associated with several viral infections, including HCV, SARS-CoV-2 and HIV. For example, during HCV infection, miR-320 is upregulated and has a strong proviral role by targeting the PI3K/Akt signalling pathway, thus extending cell survival [92], a pathway significantly downregulated by MMTV as well [44], an aspect that needs to be explored further. On the other hand, the miR-320 family is highly downregulated during severe COVID-19 and has been proposed as a biomarker for severe disease [93]. Similarly, during HIV infection, miR-320 has been proposed to be a stage-specific biomarker since it has been observed to be one of the 12 miRNAs consistently observed to be downregulated in four classes of HIV-1-infected patients [94].

In conclusion, the present study represents one of the initial investigations that provides evidence for recognition and interaction between host miRNAs and the MMTV genome. We not only identified 10 host miRNAs differentially regulated by MMTV with the ability to potentially target the MMTV genome, as supported by miRNAseq and RIP assays, but we also characterized the binding sites of three of the most affected miRNAs on the MMTV genome, and identified the genes and regulatory pathways targeted by these miRNAs. Our findings indicate that host miRNAs can selectively target the MMTV genome, thus exerting regulatory control over viral expression and its replication. Since miRNAs can act as both antiviral and proviral agents, it will be of great interest to investigate the impact of these interactions on various stages of the virus life cycle and their ability to influence mammary tumorigenesis and leukaemias/lymphomas in mice. This is important since MMTV has also been linked to breast cancer, lymphomas and other pathologies in humans [95,96]. Although highly controversial and not considered as a separate species (reviewed in [97–99]), if confirmed, our work could provide a miRNA-based framework to develop antivirals and therapeutic modalities to thwart MMTV spread and its associated neoplasms in humans.

## Data Availability

The miRNAseq data (BioProject accession number: PRJNA1000922) and mRNAseq data (BioProject accession number: PRJNA915407), both in their raw and analysed forms, are available in the electronic supplementary material. These datasets can be utilized for data reanalysis and subsequent processing. The datasets in the electronic supplementary material are grouped as follows. Supplementary Data 1: miRNA selection using the four bioinformatics tools: miRanda, STarMir, RNA Hybrid and RNA22. Supplementary Data 2: high-resolution images and associated data of the miRNA–mRNA docking analysis. Supplementary Data 3: hydrogen bonding and CPs of each miRNA on the MMTV genome from the docking analysis. Supplementary Data 4: full images of gels reported in figure 8 along with the appropriate molecular markers. Supplementary material is available online [100].
